# Cultural adaptation of the polish version of the brief pain inventory short form among the elderly

**DOI:** 10.1038/s41598-025-13132-x

**Published:** 2025-07-30

**Authors:** Iwona Repka, Piotr Brzyski, Patrycja Zurzycka, Ilona Kuźmicz, Grażyna Puto

**Affiliations:** 1 Department of Surgical Nursing, Institute of Nursing and Midwifery, Faculty of Health Sciences, Jagiellonian University Medical College, Kopernika 25 Street, Kraków, 31-501 Poland; 2 ”Dziupla” Statistical Analyses Piotr Brzyski, Aleje Jerozolimskie 85/21, Warsaw, 02- 001 Poland; 3 Department of Specialized Nursing, Institute of Nursing and Midwifery, Faculty of Health Sciences, Jagiellonian University Medical College, Kopernika 25 Street, Kraków, 31-501 Poland; 4 Department of Internal and Geriatric Nursing, Institute of Nursing and Midwifery, Faculty of Health Sciences, Jagiellonian University Medical College, Kopernika 25 Street, Kraków, 31-501 Poland

**Keywords:** Chronic pain, Non-cancer pain, Older people, Validity, Reliability, Geriatrics, Quality of life

## Abstract

Demographic changes taking place in society require a holistic approach to the health problems of the elderly, including the chronic pain that often accompanies them in various disease entities. The aim of this study was to evaluate the psychometric properties of the Polish version of the Brief Pain Inventory-Short Form (BPI-SF) among the elderly. The study was conducted on a group of 181 patients of both genders, over 65 years of age, with pain lasting longer than 6 months, hospitalized in internal medicine and geriatric wards. Construct validity was assessed using principal component analysis (PCA) method with Quartimax rotation. The goodness of fit of the measurement model, the BPI-SF was based on, in the sample of Polish elderly was tested using the Confirmatory Factor Analysis (CFA). The reliability of the scale, in terms of its internal consistency, was estimated using the Cronbach alpha and Raykow’s Rho coefficients and by analysis of item-total correlation, applying the Kline criterion. The PCA revealed unidimensional structure in the BPI-SF the first factor of the solution was defined by all but two items of the scale. The reliability of the total score was 0.88 and remained at the same level after removing sleep position and average pain intensity from the subscales. Raykow’s Rho for the individual subscales was estimated at 0.78 for severity subscale, 0.66 for affective disturbances involving sleep and 0.82 excluding it, and 0.86 for activity-related difficulties. The Brief Pain Inventory-Short Form (BPI-SF), despite differences in psychometric properties with respect to the original results, may be applicable in monitoring the intensity of chronic non-cancer pain (pain associated with the musculoskeletal system and neuropathic pain) among the elderly as well as in the verification of functional status in daily clinical practice.

Trial registration: Statutory research “Chronic pain in people over 65 years of age” K/ZDS/005733, conducted in 2015-2018.

**Supplementary Information:**

The online version contains supplementary material available at 10.1038/s41598-025-13132-x.

## Introduction

Population aging continues to be an increasingly important issue, as evidenced by World Health Organization projections that the number of people of this age will reach trillion by 2050^1,2^. In Europe, 30% of the population will be over 65 years of age by 2060^2^. In contrast, Poland is among the countries in which the percentage of residents over 60 will reach 8.5 million by 2030^3^.

The aging process predisposes individuals to disruptions in the stimulus perception process, which reduces their response to stimulation and identification in terms of sensation^4^. In addition, the risk of multimorbidity increases with age, resulting in an increase in the frequency and severity of chronic pain, with a predominance of receptor pain (58%) and mixed pain with a neuropathic component (32%)^5^. Living with chronic pain affects the quality of life of the elderly, which can manifest as: social isolation, sleep disturbances, and depression^5,6^.

Among the elderly, it is possible to distinguish real difficulties in the evaluation of pain, mainly due to the severity of cognitive impairment, which translates into problems in communication, memory, and mental health^7^. Physiological changes in the aging process play, an important role in the selection of research tools, which, in addition to unidimensional evaluation (e.g.; Visual-Analog Scale [VAS], Verbal Descriptor Scale [VDS], Faces Pain Scale [FPS]), should consider methods of coping with pain, as well as abilities in terms of daily functioning. A commonly used questionnaire that considers the above components includes the full version of the Mc- Gill Pain Questionnaire (MPQ) and Short Form Mc-Gill Pain Questionnaire (SF-MPQ)^8^. Also, into the group of multidimensional pain evaluation we will classify the Brief Pain Inventory-Short Form (BPI-SF) identified by Cleeland^9^, with its scope covering not only the intensity of pain and its nature, but also its impact on functional status. It is a key diagnostic tool for verifying ongoing treatment in patients experiencing chronic pain^10^.

As most of the measurement scales created to measure so called latent variables is used in different cultural conditions than they were created, it is very important to properly conduct the process of cross-cultural adaptation to obtain the measurement tool which will retain the psychometric properties of the original tool in new country. The process consists of two stages: the translation process including forward and backward translation, and the validation study – examination of the version of the measurement tool created during translation – leading to estimation of the validity and reliability of the created tool. The validity problem relates to question whether the tool measures the same latent variables it did in original conditions, and reliability refers to the precision of the measurement, in term of the impact of the measurement error on the observed score^11,12^.

The aim of this study was to evaluate the psychometric properties of the Polish version of the Brief Pain Inventory-Short Form (BPI-SF) among older people suffering from chronic non-cancer pain.

## Methods

The study was conducted among 181 patients hospitalized in the Internal Medicine and Gerontology Department of the University Hospital and the Military Clinical Hospital, as well as in the Health Care Center of the Ministry of Internal Affairs and Administration in Cracow. A total of 200 patients were eligible for the study, although five patients were excluded during the study due to incomplete responses and 14 patients due to refusal to participate in the study. The sample size was estimated to fulfill the rules most often found in the literature, talking finally into account the most demanding of them. A long-standing rule it thumb found in the literature is that sample size should be at least 50 more than 8 times the number of variables in the model, which in case of our model leads to number of at least 162^13^. Moreover Bentler and Chou^14^ suggested at least 5 cases per estimated parameter resulting in number of at least 125 cases, whereas Stevens^15^ indicated at least 15 cases per indicator, resulting in at least 165 cases. We also aimed to fulfill a criterion suggested by Loehlin^16^ and Hoyle^17^ that sample should be of at least 100 to 200 cases.

The inclusion criteria for the study were as follows: age ≥ 65 years, chronic pain of various origins, persisting for at least 6 months, written consent from the patient to participate in the study, no cognitive impairment - verified with the Abbreviated Mental Test Score (AMTS) (obtaining a value above 6 points), and no diagnosis of malignant disease. The data collection process included a demographic data characterization form (age, sex, educational level, place of residence, and marital status) and the Brief Pain Inventory-Short Form (BPI-SF). Individuals who met the study criteria were informed verbally and in writing by the researcher about the purpose and course of the study, including a discussion of the research tools used, as well as the possibility of withdrawing from the study at any time without any consequences. Each respondent was asked to select one answer from the options provided for each question. The study lasted approximately 15 min. The coordinator and/or a member of the research team was present with the respondent throughout the study to provide assistance.

The Brief Pain Inventory-Short Form (BPI-SF) was developed in English and subjected to component validity and internal consistency analysis by Cleeland in 1991, and it is one of the primary and recommended tools for pain evaluation through the Initiative on Methods, Measurement and Pain Assessment in Clinical Trials (IMMPACT)^18^. The Brief Pain Inventory-Short Form (BPI-SF) is a multidimensional tool used to evaluate pain and its effects on various life aspects. The first question was designed to screen for verify the type of pain on the day of the evaluation. The next area of the scale consisted of an image of the human body in the frontal plane from the front and back (Question 2), on which respondents could mark where they felt pain. It should be noted that there are no limits to marking the painful areas. The remaining questions were based on an numerical 11-point scale where 0 indicated no pain and 10 indicated the worst pain perceived. In questions 3, 4, and 5, the scale covered the intensity of pain in the last 24 h preceding the survey, while question 6 referred to the time of the survey. One open-ended question was included in the BPI scale, which provided an option to list the analgesic pharmacology and treatment modalities used. One of the questions (number 8) was related to pain relief (verification based on a numerical scale, where the changes taking place are expressed in percentages). That 0 indicates no pain relief, 100% indicates complete pain relief). Question 9 verified the effect of pain on functional status, including: general activity, mood, relationships with others, sleep, movement ability, professional work and activities performed at home, and enjoyment of life. This question also uses an 11-point scale to monitor the changes taking place, with extreme answers such as 0 indicating no impact on a particular activity/life activity, and 10 indicating total impediment in a particular area^19^.

In the present study, after receiving the authors’ permission to use the tool in the project presented here, the original version of the BPI-SF was translated using the forward - backward method from English to Polish by two independent interpreters experienced in translating medical texts. The obtained versions were compared in terms of content, and discrepancies between translations were verified to select the most appropriate version of the Polish translation of the BPI-SF scale. The next stage involved backward translation, which consisted of two independent translators translating the received version of the BPI-SF from Polish to English. Both versions were compared with the original version to eliminate possible inconsistencies^18,19^. In the process of cross-cultural adaptation, acceptability was a key element ensuring the correctness of translations and their usefulness in the target cultural circle. Acceptability was verified through cognitive debriefing interviews, which allowed respondents to understand and interpret individual questions and indicate areas for minor modifications. In addition, expert opinions were used to verify the translations, mainly in the cultural and linguistic context, thus enabling the evaluation of the accuracy and adequacy of the content of the BPI-SF scale^20^.

The selected version of the Polish translation of the BPI-SF scale (see Supplementary material 1) was subjected to the process of psychometric validation within the statutory research “Chronic pain in people over 65 years of age” (K/ZDS/005733) from 2015 to 2018, for which approval was obtained from the Jagiellonian University Bioethics Committee (KBET/83/B/2013). All participants provided informed consent prior to inclusion in the study. The study protocol was written and conducted in accordance with the Declaration of Helsinki. All team members were trained to introduce, show, and address questions regarding informed consent and the assessments to be performed before participants provided informed consent.

### Statistical analysis

The construct validity of the BPI-SF was investigated using exploratory factor analysis (EFA) to assess its factor structure in polish sample. This was performed using the Principal Component Analysis (PCA) method with Quartimax rotation^21,22^. The number of extracted factors was determined based on the criteria of eigenvalues greater than 1 and interpretability of the factors.

**Fig. 1:**
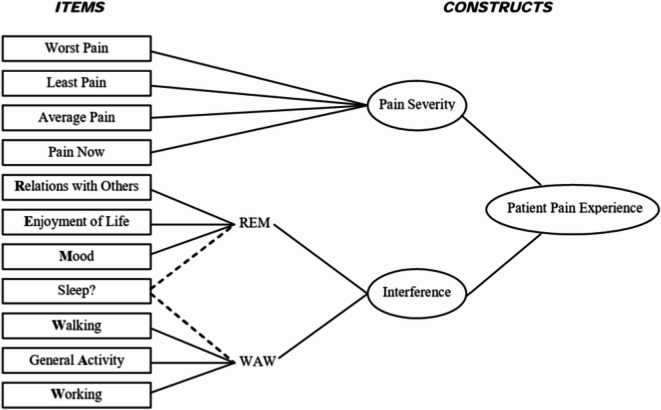
Confirmatory factor analysis (CFA) of BPI-SF for two-factor model proposed by Cleeland (1991, 2009).

To test how well the theoretical model of the BPI-SF (as proposed by Cleeland)^9,23^ fits the data collected from a sample of Polish elderly people hospitalized in non-surgical wards and reporting chronic non-cancer pain with different degrees of severity lasting more than 6 months, a confirmatory factor analysis (CFA) was conducted. Cleeland^9,23^ stated that the BPI-SF has a two-dimensional structure consisting of severity and interference dimensions, but he also distinguished between two subdimensions of the interference dimension: affect (REM) and activity (WAW) (see Fig. 1).

Therefore, we tested three models: the 1 st model (the baseline model) assumed that severity, affect, and activity constitute three correlated, but not identical, factors (i.e. correlations between factors are not constrained in the model). Additionally, as Cleeland^9,23^ did not give a clear statement as to which subdimension of the interference the sleep item belonged, we modified the 1 st model by restraining the path coefficients so that this item could belong only to activity (model 1a) or to the affect subscale (model 1b). The 2nd model assumed that activity and affect factors were the same factor (correlation between them was constrained to equal 1), which means that the BPI-SF is a two-dimensional construct, whereas the 3rd model assumed that the BPI-SF is a unidimensional construct (correlations between all pain dimensions are constrained at 1).

The goodness of fit of the models was evaluated based on the root mean square error of approximation (RMSEA)^24^ a population-based index, that measures the discrepancy per degree of freedom, which is relatively insensitive to sample size. The goodness of fit of the model was also analyzed using the Goodness of Fit Index (GFI)^25^ and Adjusted Goodness of Fit Index (AGFI)^25^. Additional goodness-of-fit indices, such as chi-square statistics, Normed Fit Index (NFI)^26^, and Comparative Fit Index (CFI)^27^, were inspected to provide additional evidence of the model fit. Based on recommendations from prior Monte Carlo studies^28^, the goodness-of-fit statistics were evaluated using arbitrary cutoffs of less than 0.08 as an acceptable fit, and a goodness of fit of RMSEA. We expected that the PCLOSE measure (probability that the population RMSEA was less than or equal to 0.05) would exceed 0.05^29^. It was expected that chi^2^ statistics would not be significant and that chi^2^ divided by the number of degrees of freedom would not exceed five for well-fitted models. For the other indexes a value of at least 0.90 was considered an adequate fit, whereas 0.95 was considered a good fit.

The reliability of the subscales and the total score, in terms of their internal consistency, was tested using the Cronbach’s alpha coefficient^22,30^ and by analysis of item-total correlation, applying the Kline criterion. It was expected that correlations between the total scale score and the subscales, and their items would exceed 0.4^31^. Alpha coefficients were also estimated using Raykov’s rho coefficient^22,32^, as some authors have raised concerns that alpha coefficients often underestimate or overestimate true reliability. Values of alpha and rho coefficients higher than 0.7 were considered acceptable, while those higher than 0.8 were considered satisfactory.

The EFA and reliability analyses were conducted using the IBM SPSS Statistics 29 statistical package, whereas the CFA was conducted using IBM SPSS AMOS 29 statistical software.

## Results

In the sample analyzed, the participants ages ranged from 65 to 98 years. The average age of the respondents was 77. The proportion of female respondents was higher than the proportion of male respondents (61.9% vs. 38.1%). The largest group comprised respondents with a high school education (47.5%). Those living in an urban environment were dominant (70.2%). Almost half of the respondents were divorced or widowed (48.1%) (Table 1).

**Table 1:** Sociodemographic characteristic of the study sample.

Sociodemographic characteristics	Mean	SD
Age (years)	77.1	7.9
Gender	N	%
women	112	61.9
men	69	38.1
Education	N	%
primary	20	11
vocational	36	20
secondary	86	47.5
university	39	21.5
Place of residence	N	%
urban areas	127	70.2
countryside	54	29.8
Marital status	N	%
married	81	44.8
widowed or divorced	87	48.1
single	13	7.2

N - number of respondents; % - percentage of respondents; Mean; SD - standard deviation.

The factor structure of the BPI-SF, revealed in the EFA in our study, differed significantly from the theoretical structure proposed by Cleeland^9,23^. PCA was conducted for 11 items constituting the BPI-SF in a sample of Polish elderly people hospitalized in non-surgical wards, and reporting chronic non-cancer pain with different degrees of severity, lasting for more than 6 months, revealed three components with eigenvalues exceeding 1, which together explained 74% of the total variance of the items (46%, 17%, and 11%, respectively). In the non-rotated solution, all items, except two, loaded on 1 st component at a level higher than 0.6, and these loadings had the highest loadings of these variables. Therefore, it can be stated that the BPI-SF has a unidimensional structure in the elderly Polish sample. One of the other two items, - average pain - loaded on the 1 st factor at the level of 0.59, but on the 3rd factor it loaded at the level of 0.71; -thus, it can be stated that it constituted the third factor itself. The item with the lowest loading on the 1 st component - “pain affected the sleep” - had slightly higher loading on the 2nd component (see Table 2).

**Table 2:** Factor structure of the BPI-SF in the sample of Polish elderly.

	Non-rotated 3-factor solution			Quartimax-rotated 3-factor solution		
	1	2	3	1	2	3
P3. Worst pain	0.66			**0.69**		
P4. Least pain	0.66	−0.57			**0.82**	
P5. Average pain	0.59		0.71			**0.81**
P6. Pain at the moment	0.66	−0.50			**0.76**	0.41
P9A. General activities.	0.74	0.45		**0.88**		
P9B. Mood	0.71	−0.47	−0.41		**0.84**	
P9C. Locomotion	0.76			**0.80**		
P9D. Work	0.78			**0.80**		
P9E. Relationships	0.75	−0.46			**0.84**	
P9F. Sleep	0.44	0.51		**0.59**		0.42
P9G. Enjoyment of life	0.72			**0.78**		

Factor loadings lower than 0.4 are hidden.

Application of Quartimax rotation revealed that the BPI-SF in this sample had a three-dimensional structure, with the 1 st dimension defined by five items related to the interference of pain (the total activity subscale, sleep, and enjoyment of life items). Notably among the severity items, only the most severe pain was loaded on this factor. The 2nd dimension was defined by two other items of the affect subscale, as well as by items concerning the lowest pain and current pain. The 3rd factor, in both rotated and non-rotated solutions, consisted exclusively of the average pain item. Attempts to reproduce the factor structure proposed by the authors of the scale by limiting the number of factors to two provided results that were similar to the 3-factor solution. The only difference concerned the average pain item, which in the 2-factor solution had similar loadings on both factors in the two-factor solution (see Table 2).

**Fig. 2:**
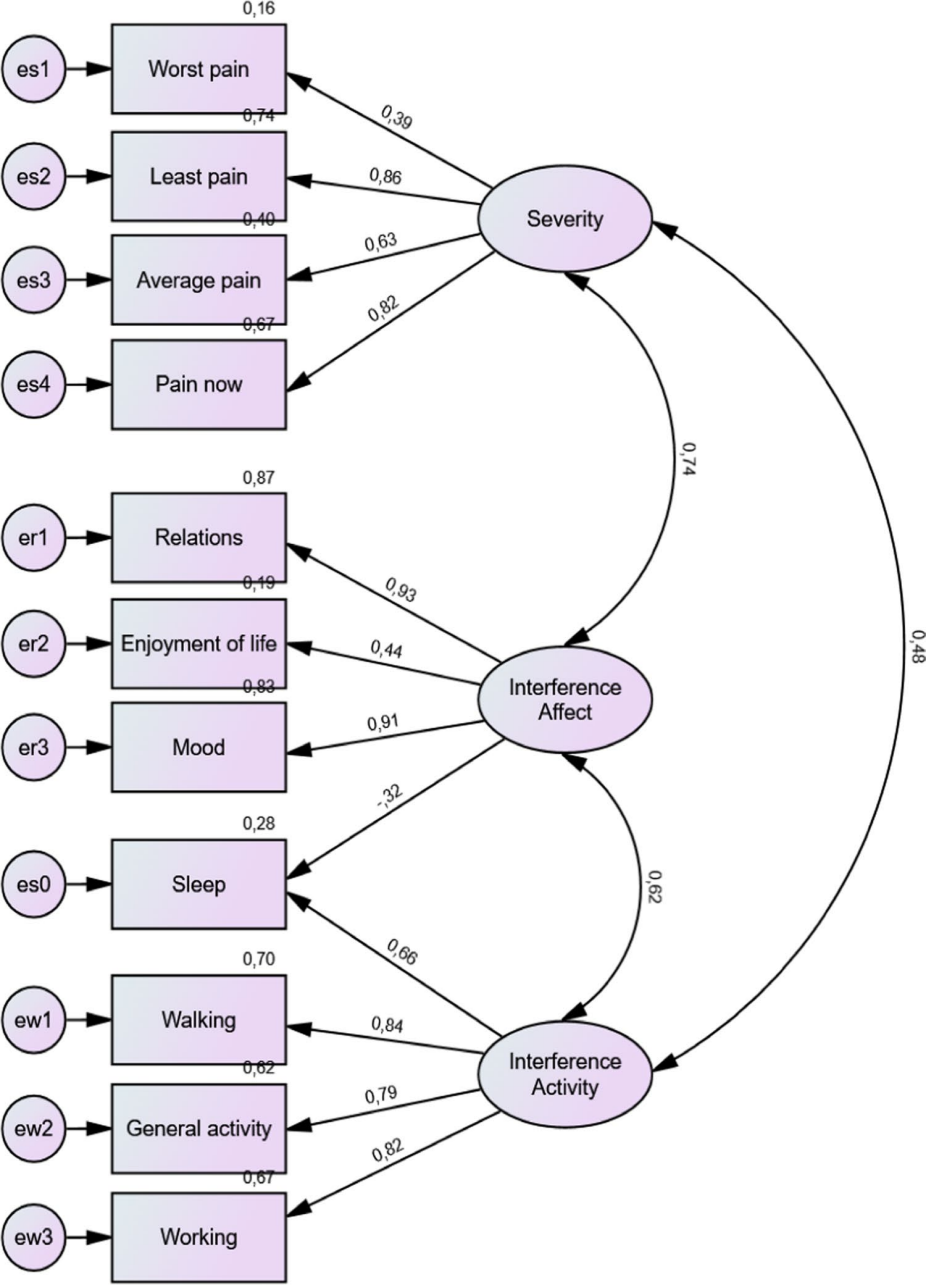
Confirmatory factor analysis (CFA) for BPI-SF in the Polish elderly sample.

We used CFA to test whether the theoretical factor structure of the BPI-SF, as defined by Cleeland^9,23^, fits the data collected in a Polish sample of elderly hospitalized in non-surgical wards and reporting chronic non-cancer pain with different degrees of severity, lasting for more than six months. The figure below shows the path diagram of CFA for the BPI-SF in the Polish elderly population in Model 1 (see Fig. 2). The ovals are the latent variables measured by the model, and the rectangles represent the observed indicators. The numbers placed above the upper-right corner of the rectangles are the coefficients of determination in the regression of the indicator on the latent variable it represents. Numbers placed above one-directional straight arrows, leading from ovals representing latent variables to their indicator, are the factor loadings – standardized regression coefficients of the indicator on the latent variable. The numbers above the two-directional arch arrows are the correlation coefficients between the latent constructs. To ensure model identification, the variance of the latent variables was set to 1.

The best-fitting model was Model 1 (the baseline model). However, the value of the chi^2^ statistics for this model was significant, indicating that the model did not fit the data well. Additionally, the quotient of the chi^2^ statistics divided by the number of degrees of freedom exceeds five, which is considered a liberal criterion for well-fitted models. The chi^2^ statistics are known to easily reject the hypothesis that the data do not fit the model well; -thus, other fit indices should be investigated. However, they did not indicate satisfactory goodness of fit. The GFI and AGFI for the best -fit model reached values of 0.73 and 0.56 respectively. NFI and CFI indexes obtained similar values as GFI index (0.73 and 0.74, respectively). RMSEA reached a value of 0.21, which is significantly higher than the level of 0.05.

Both models, which assumed that sleep items belong to only one of the interference subscales, had a significantly worse fit than the baseline model, which assumed that this item belongs to both subscales (1a and 1b). Model 2, which assumed that all interference items form one dimension, and Model 3, which assumed that pain is an unidimensional construct (3), also had significantly worse goodness-of-fit than the baseline model (1).

The reliability of the total score and of subscales was estimated based on Cleeland theoretical model^9,23^. The reliability of the severity scale was estimated at a level of 0.77, and only the item with the strongest pain, when excluded from the scale, caused an increase in Cronbach’s alpha of 0.81. For the other items, exclusion of the item decreased the alpha value to approximately 0.68. The alpha value estimated for the affect interference subscale, including the sleep item, reached a reliability of 0.70 and only the sleep item caused an increase in the alpha value to a level of 0.78, when removed from the subscale. The removal of any of the other items decreased the alpha to levels ranging from 0.51 0.62. The alpha value estimated for the activity interference subscale, including the sleep item, reached a reliability of 0.81, and only the sleep item - similar to the affect subscale, - caused an increase in the alpha value to 0.85, when removed from the subscale. Removing any other items decreased the alpha to 0.70 and 0.74. The reliability of the interference subscale reached 0.86, which increased to 0.87 only when the sleep item was excluded. When any of the other items was removed from the subscale, reliability decreased to a level ranging from 0.82 to 0.85. The reliability of the total BPI-SF score reached 0.88, which remained at 0.88 only when the sleep and average pain items were removed from the subscale. When one of the other items was removed from the subscale, reliability decreased to a level between 0.86 and 0.87.

Raykov rho for the respective subscales was estimated at a level of 0.78 for severity, 0.66 for affect interference including sleep and 0.82 excluding it, and 0.86 for activity interference, both including and excluding sleep. Raykov rho for interference subscale was estimated as equal to 0.85, including sleep item, and 0.87, excluding this item, and for the total score it was estimated at level of 0.87 (including sleep) and 0.88 (excluding sleep).

For the severity subscale, all items correlated with the total score at a level ≥ 0.4, except for the most severe pain, which correlated with the total score at a level of 0.39. All items from the affect interference subscales, except sleep (*r* = 0.20), met Kline’s criterion, while in the case of the activity interference subscales, all items, including sleep (although its correlation was the weakest among these items), met this criterion, correlating with the total score of the subscales at a level higher than 0.4. Regarding the interference subscale and the total score of the BPI-SF, in both cases the sleep item was the only one that did not meet Kline’s criterion (0.34 and 0.35, respectively).

## Discussion

The present study, which investigated the psychometric properties of the BPI-SF in the Polish sample of elderly people reporting chronic non-cancer pain with different degrees of severity, lasting for more than six months, revealed results that were only partially similar to those presented by other authors. The revealed factor structure differed significantly from that proposed by Cleeland^9,23^, who distinguished three dimensions: one consisted of items related to the perception of pain severity, and two others consisted of items related to the interference of physical (activity - WAW) and emotional (affect - REM) domains of everyday life.

However, Cleeland^9,23^ in his study reported psychometric properties from studies conducted in 4 countries: the United States, Mexico, Philippines, and Vietnam. In all of them a 2-factor structure was obtained. He stated that: for each language version, the same two factors emerged with an eigenvalue greater than 1: the first factor comprised the pain interference items and the second factor comprised the pain severity items. He also concluded that: the similarity of the factor loadings among the language versions indicated that cancer and pain patients living in different countries and speaking different languages responded similarly to the items^9,23^.

Other authors, who also conducted their studies in samples of cancer patients, reported almost identical factor structures as the scale authors. Similar results were reported by Nakamura, who conducted studies in a sample of 121 adult patients (aged 19–82 years, mean age 56 years), who had a pathological diagnosis of cancer and also experienced pain, and 86% of them were receiving active pain treatment^33^. Similar results were obtained by Leppert and Majkowicz^34^, who conducted a study of 30 adult patients participating in a clinical trial (age, 48–83 years; mean age, 70 years). They were opioid-naive patients with nociceptive (visceral or somatic) cancer pain who were treated with either tramadol or DHC^34^.

The factor structure, which was very similar to that described by the scale authors and differed only in the order of factors, was confirmed by other authors who also conducted their studies on several samples of cancer patients^19,35^. The study by Aisyaturridha et al.^35^ included 113 adult patients (age 18–76 years, mean age 46 years), who were diagnosed with any type of cancer and experienced pain 24 h prior to the interview, with or without any analgesic treatment^35^. A greater number of participants were included in the Koios study^36^, with approximately 250 respondents having chronic pain persisting for more than 3 months, over 65 years of age, predominant lower back pain (82.4%), and degenerative joint changes (72.8%). Majedi et al.^19^ conducted a study of 201 adult patients from a pain clinic (aged ≥ 18 years), with malignant and non-malignant conditions and, chronic pain for more than three months.

In addition, Ferreira et al.^37^ and Alizadeh-Khoei et al.^38^ reported that the highest pain intensity was observed within a short interval during 24-hour verification. This indicates that pain intensity evaluation is susceptible to interference factors. The psychometric properties of the BPI-SF in a sample of elderly people with chronic nociceptive and neuropathic pain showed average pain intensity and inferential results, demonstrating excellent reliability and dependability in multidimensional evaluation.

The factor structure most similar to the theoretical factor structure proposed by Cleeland^9,23^, was obtained by Klepstad et al.^39^ The author conducted this study in a sample of 300 adult patients (age, 29–89 years; mean age, 63 years), with malignant disease who received regular morphine treatment^39^. He obtained a 3-factor structure with factors defined by activity, affect (including sleep), and severity items. Our factor structure also consisted of three factors, but the 3rd one was defined by one item: - average pain. The 1 st factor of our solution was defined by all activity items, one affect item, the most severe pain item, and one sleep item. The differences between factor structures may result mainly from the differences between samples; our sample consisted of elderly patients (aged 65–98 years, mean age 77 years), hospitalized in non-surgical wards, and reporting chronic non-cancer pain with different degrees of severity, lasting for more than 6 months. In all studies, except Majedi et al. sleep had the weakest loading on the respective factor, which is consistent with the opinion of the scale authors, who could not unambiguously ascribe this neither to the activity or to the affect dimension^19^.

Our results suggest that, in older people, average pain is a general measure of pain and its impact on different aspects of daily life. However, the most severe pain seems to be strongly related to older adult’s performance in daily life, especially in different aspects of physical activity. On the other hand, average and current pain seem to be measures of the impact of pain on emotional aspects of daily life, including relationships with others.

CFA showed that according to all goodness-of-fit coefficients, the theoretical model proposed by Cleeland^9,23^ does not fit our data well. The main reason for this is the difference between factor structures resulting from differences in the correlation matrix between all items of the scale, but also the diversity in the range of path coefficients for indicators of the same dimension. These differences probably results from the differences between the ages of patients, and, to a lesser extent, from the fact that Polish patients were not cancer patients. All other authors, whose results were reported in this study, conducted their studies in samples of adult patients, and only in Leepert and Majkowicz^34^ did the lower limit of the age of patients be significantly higher than 18 years. Adult patients from the cancer samples were mainly fully independent patients regarding their daily activities, and their pain originated from their main health problem - cancer. The adult patients in the cancer sample group were mainly fully independent in terms of their daily activities, and their pain originated from their primary health issue - cancer. In the elderly group, additional disturbances in pain perception were most likely caused by the location of pain in a larger part of the body, which may be due to fatigue and additional burdens associated with frailty syndrome. In contrast, in the elderly patients included in our sample, pain is a consequence of the natural aging process and can result from both the degeneration of the organism itself and from diseases that are the consequence of aging or that accompany this process. It is important to emphasize that the ability to cope with chronic pain decreases with age, which may contribute to cognitive impairment. This is particularly relevant for the impairment of selective attention, which is associated with inhibition of distracting stimuli. Moreover, reduced attentional capacity in the elderly impairs cognitive function and sensory information processing, which affects attitudes toward pain, as well as balance disorders, thereby impairing mobility and increasing the risk of falls^40^.

The differences between the factor structures resulted in differences in subscale reliability. The reliability, measured by Cronbach alpha, was estimated at the level of 0.77 and, measured by Raykov rho, at 0.78 for the severity dimension and at 0.86 and 0.85 for the interference dimension, respectively. Regarding the subscales of the interference dimension, coefficients of 0.70 and 0.66 were obtained for affect and 0.81 and 0.85 for the activity subscales, respectively. Cleeland^9^ reported psychometric properties from studies conducted in 4 countries ranging from 0.80 to 0.87 for severity and from 0.89 to 0.92 for interference. Similar values of reliability coefficients – ranging from 0.86 to 0.88 for severity and from 0.91 to 0.92 for interference – were reported by some of other authors mentioned above Majedi et al.^19^, Leppert and Majkowicz^34^, Klepstad et al.^39^. A slightly lower reliability was reported by Aisyaturridha, who estimated the reliability of the severity at 0.81 and for interference at 0.88^35^. Only one author, among those who conducted their studies in samples of cancer patients, estimated the reliability of the BPI at a level similar to than in our study: Uki et al.^33^ reported an internal consistency reliability of 0.81 for both subscales.

Estimations of the reliability of the subscales based on our sample wear significantly lower than those in other studies. It is consistent with different factor structure than obtained by other authors, and – to some extent – inconsistent with goodness of fir of the CFA model, which was far from being acceptable. However in spite of that, intercorrelations between items included in particular BPI-SF dimensions remained high enough to provide satisfactory level of reliability. Regarding the severity dimension, it follows that its items are loaded on all three factors extracted by the PCA procedure. Considering the interference subscale, the items were loaded on two of the extracted factors. In our study, the sleep item loaded on the same factor, where all activity items loaded highly, but sleep item loading was the lowest. In addition, in the non-rotated solution, the loading of the first factor was significantly lower than that of the other items. It is concerned with results of reliability analysis which showed that in case of all subscales we tried to include the sleep item, it caused increase of value of alpha coefficient when it was removed from the scale. The sleep items did not meet Kline’s criteria for all subscales. In addition, the results of the CFA showed that it was difficult to include sleep items in any of the subscales to improve the psychometric properties of the subscale. This may suggest that the sleep item measures another dimension of pain perception in elderly and its measurement should be extended by adding additional item concerning the sleep.

## Conclusions

The factor structure of the BPI-SF in the elderly population differs from that established in the cancer patient population. However, the reliability of the subscales proposed by authors of the scale reached mostly acceptable level. The BPI-SF is a useful tool for monitoring chronic pain of diverse nature in a group of elderly individuals in daily practice. Lack of studies conducted on other elderly populations prevents strong conclusions from being drawn based on the results of a single study.

Limitations of the study were related to its cross-sectional nature, which did not reflect changes occurring in relation to multimorbidity and the pain management methods used. The study is limited to hospitalized patients, potentially reducing generalizability to community-dwelling elderly. The study focused generally on individuals over 65 years of age without comparisons between age groups in this group. Future studies should focus on the verification of the nature of chronic pain during the selection of study groups and the pharmacological and non-pharmacological treatment used in older individuals.

## Electronic supplementary material

Below is the link to the electronic supplementary material.

Supplementary Material 1

## Data Availability

Availability of data and materialsThe datasets analyzed during the current study are available from the corresponding author on reasonable request.
