# Supplementary material for: Cultural adaptation of the polish version of the brief pain inventory short form among the elderly
Source: Sci Rep. 2025 Jul 30;15:27885. doi: 10.1038/s41598-025-13132-x (PMC12311105; doi:10.1038/s41598-025-13132-x)
Supplement: Supplementary file 1 — Supplementary Material 1 [file 41598_2025_13132_MOESM1_ESM.doc]

**I am sending the Polish version in the supplementary file section, because it is a cultural adaptaion in the Brief Pain Inventory - Short Form (BPI-SF).**

# Supplementary material

Krótki Inwentarz Bólu (Polish Version)

Data: Godzina:

Nazwisko i imię: Inicjały:

1. Podczas życia, większość z nas od czasu do czasu odczuwa ból (taki jak ból głowy, zwichnięcie stawu, ból zęba). Czy odczuwał Pan / i inny niż wymienione rodzaje bólu dzisiaj ?

1. Tak 2. Nie

1. Proszę zakreślić na rysunku obszar, w którym odczuwa Pan / i ból. Proszę zaznaczyć znakiem „X” obszar, gdzie ból jest najsilniejszy.


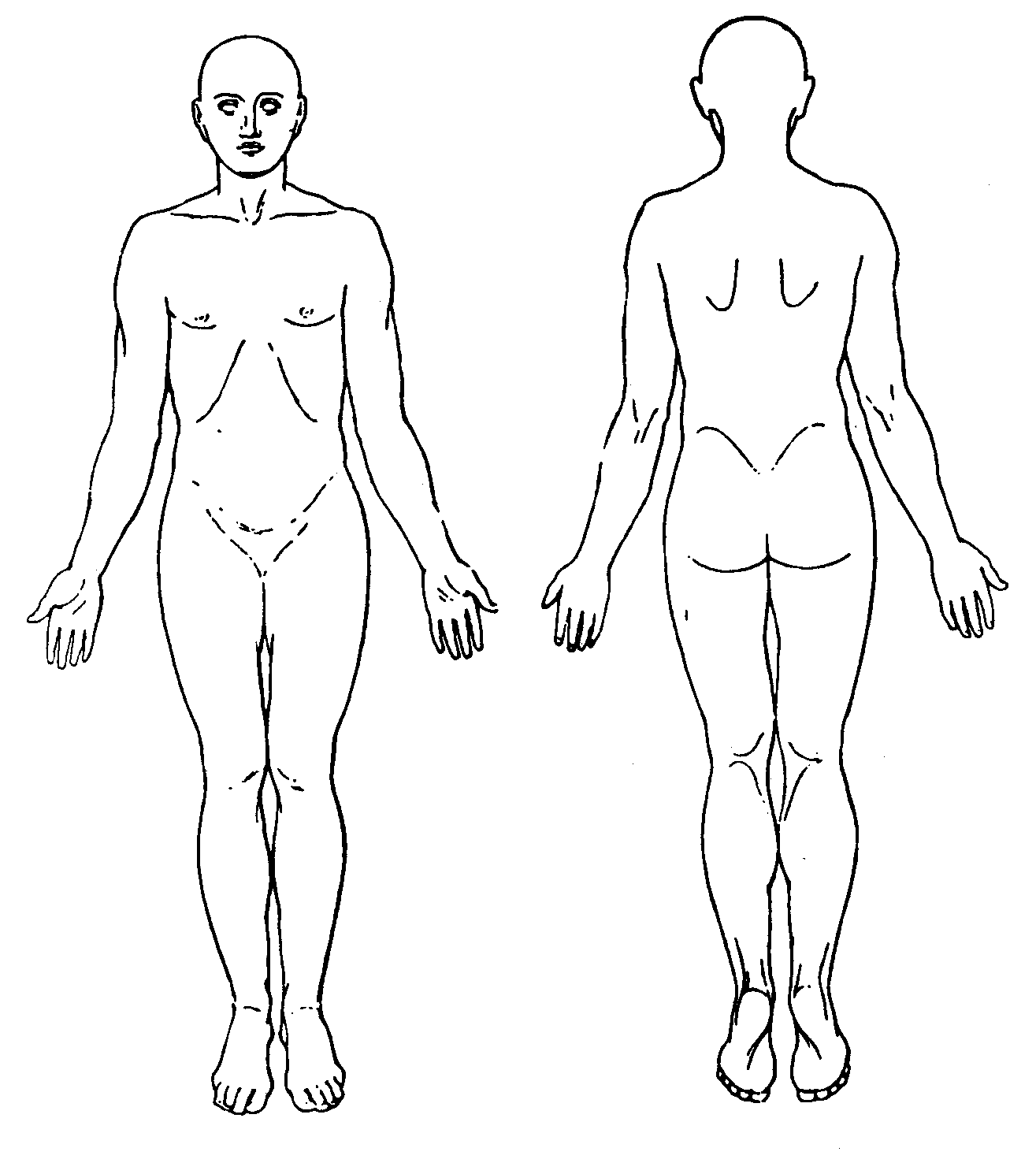


Lewa Lewa

Prawa

Prawa

1. Proszę ocenić ból poprzez zakreślenie jednego numeru, który najlepiej opisuje Pana /i ból, kiedy był najsilniejszy, w ciągu ostatnich 24 godzin

0 1 2 3 4 5 6 7 8 9 10

Brak Ból najsilniejszy jaki

bólu można sobie wyobrazić

1. Proszę ocenić ból poprzez zakreślenie jednego numeru, który najlepiej opisuje Pana / i ból, kiedy był najsłabszy, w ciągu ostatnich 24 godzin

0 1 2 3 4 5 6 7 8 9 10

Brak Ból najsilniejszy jaki

bólu można sobie wyobrazić

1. Proszę ocenić ból poprzez zakreślenie jednego numeru, który najlepiej opisuje jak silny średnio był Pana / i ból

0 1 2 3 4 5 6 7 8 9 10

Brak Ból najsilniejszy jaki

bólu można sobie wyobrazić

1. Proszę ocenić ból poprzez zakreślenie jednego numeru, który mówi jak silny jest Pana /i ból w chwili obecnej

0 1 2 3 4 5 6 7 8 9 10

Brak Ból najsilniejszy jaki

bólu można sobie wyobrazić

1. Jakie sposoby leczenia lub jakie leki otrzymuje Pan / Pani z powodu bólu?

8. W ciągu ostatnich 24 godzin, jak dużą ulgę w bólu spowodowało leczenie bólu lub podawane leki? Proszę zakreślić jedną liczbę w procentach, która to najlepiej pokazuje

0% 10% 20% 30% 40% 50% 60% 70% 80% 90% 100%

## Brak Całkowita

ulgi ulga

1. Proszę zakreślić jeden numer, który opisuje jak, w ciągu ostatnich 24 godzin, ból przeszkadzał Pana/i:

A. Ogólnej aktywności

0 1 2 3 4 5 6 7 8 9 10

Nie Całkowicie

przeszkadzał przeszkadzał

B. Nastrojowi

0 1 2 3 4 5 6 7 8 9 10

Nie Całkowicie

przeszkadzał przeszkadzał

C. Zdolności chodzenia

0 1 2 3 4 5 6 7 8 9 10

Nie Całkowicie

przeszkadzał przeszkadzał

D. Normalnej pracy (zarówno poza domem, jak i pracy domowej)

0 1 2 3 4 5 6 7 8 9 10

Nie Całkowicie

przeszkadzał przeszkadzał

E. Relacjom z innymi ludźmi

0 1 2 3 4 5 6 7 8 9 10

Nie Całkowicie

przeszkadzał przeszkadzał

F. W spaniu

0 1 2 3 4 5 6 7 8 9 10

Nie Całkowicie

przeszkadzał przeszkadzał

G. Korzystaniu z życia

0 1 2 3 4 5 6 7 8 9 10

Nie Całkowicie

przeszkadzał przeszkadzał

# Bardzo dziękujemy za wypełnienie kwestionariusza !
